# In-depth analysis of ChatGPT’s performance based on specific signaling words and phrases in the question stem of 2377 USMLE step 1 style questions

**DOI:** 10.1038/s41598-024-63997-7

**Published:** 2024-06-12

**Authors:** Leonard Knoedler, Samuel Knoedler, Cosima C. Hoch, Lukas Prantl, Konstantin Frank, Laura Soiderer, Sebastian Cotofana, Amir H. Dorafshar, Thilo Schenck, Felix Vollbach, Giuseppe Sofo, Michael Alfertshofer

**Affiliations:** 1 Department of Oral and Maxillofacial Surgery, Charité – Universitätsmedizin Berlin, Corporate Member of Freie Universität Berlin, Humboldt-Universität Zu Berlin, and Berlin Institute of Health, Berlin, Germany; 2 Department of Plastic Surgery and Hand Surgery, Klinikum Rechts Der Isar, Technical University of Munich, Munich, Germany; 3 Division of Plastic Surgery, Department of Surgery, Brigham and Women’s Hospital, Harvard Medical School, Boston, MA USA; 4 Department of Otolaryngology, Head and Neck Surgery, School of Medicine, Technical University of Munich (TUM), Munich, Germany; 5 Department of Plastic, Hand and Reconstructive Surgery, University Hospital Regensburg, Regensburg, Germany; 6 Ocean Clinic, Marbella, Spain; 7 University Hospital Regensburg, Regensburg, Germany; 8 Department of Dermatology, Erasmus Medical Centre, Rotterdam, The Netherlands; 9 Centre for Cutaneous Research, Blizard Institute, Queen Mary University of London, London, UK; 10 Department of Plastic and Reconstructive Surgery, Guangdong Second Provincial General Hospital, Guangzhou, Guangdong Province, China; 11 Department of Surgery, Emory University School of Medicine, Atlanta, GA USA; 12 Private Practice, Munich, Germany; 13 Department of Hand, Plastic and Aesthetic Surgery, Ludwig-Maximilians-University Munich, Munich, Germany; 14 Instituto Ivo Pitanguy, Hospital Santa Casa de Misericórdia, Pontifícia Universidade Católica Do Rio de Janeiro, Rio de Janeiro, Brazil; 15 Department of Oromaxillofacial Surgery, Ludwig-Maximilians-University Munich, Munich, Germany

**Keywords:** ChatGPT, USMLE, USMLE Step 1, OpenAI, Medical Education, Clinical Decision-Making, Computational models, Machine learning, Patient education

## Abstract

ChatGPT has garnered attention as a multifaceted AI chatbot with potential applications in medicine. Despite intriguing preliminary findings in areas such as clinical management and patient education, there remains a substantial knowledge gap in comprehensively understanding the chances and limitations of ChatGPT’s capabilities, especially in medical test-taking and education. A total of n = 2,729 USMLE Step 1 practice questions were extracted from the Amboss question bank. After excluding 352 image-based questions, a total of 2,377 text-based questions were further categorized and entered manually into ChatGPT, and its responses were recorded. ChatGPT’s overall performance was analyzed based on question difficulty, category, and content with regards to specific signal words and phrases. ChatGPT achieved an overall accuracy rate of 55.8% in a total number of n = 2,377 USMLE Step 1 preparation questions obtained from the Amboss online question bank. It demonstrated a significant inverse correlation between question difficulty and performance with r_s_ = -0.306; p < 0.001, maintaining comparable accuracy to the human user peer group across different levels of question difficulty. Notably, ChatGPT outperformed in serology-related questions (61.1% vs. 53.8%; p = 0.005) but struggled with ECG-related content (42.9% vs. 55.6%; p = 0.021). ChatGPT achieved statistically significant worse performances in pathophysiology-related question stems. (Signal phrase = “what is the most likely/probable cause”). ChatGPT performed consistent across various question categories and difficulty levels. These findings emphasize the need for further investigations to explore the potential and limitations of ChatGPT in medical examination and education.

## Introduction

ChatGPT as the most prominent artificial intelligence (AI)-powered chatbot has arisen public and scientific interest as a versatile automatization tool and pocket encyclopaedia. Recent studies have explored the integration of AI-powered technologies across various facets of healthcare and medicine, including automated disease classification, clinical management, as well as medical and patient education^1–6^. The phalanx of chatbots such as ChatGPT represents the next generation of AI technology integrating the various capabilities of different AI algorithms. Besides round-the-clock availability and high cost efficiency, such chatbots have the ability to learn from user interactions, thereby improving and adapting to individual user preferences and requirements over time^7,8^.

Recent studies analyzing ChatGPT’s performance in the United States Uniform Bar Examination (UBE) and the United States Medical Licensing Exam (USMLE) reported surprising outcomes: Katz et al. showed ChatGPT aced the UBE with a score of 297 points nearing the 90^th^ percentile of test-takers^9^. Kung et al. used a limited set of freely accessible test questions (USMLE Sep 1: 119; USMLE Step 2CK: 102; USMLE Step 3: 122) and demonstrated that ChatGPT achieved performance levels at or near the passing threshold of approximately 60% for all three steps^10^. The ability of ChatGPT to approach the passing threshold of approximately 60% has further been substantiated by Yaneva et al. who of note also reported substantial performance variations when querying the chatbot multiple times, thereby “underscoring the need for expert validation”^11^. The standardized USMLE Steps are considered pivotal factors in the application and selection process for matching in a residency program. These scores serve as the primary objective and quantifiable metric, thereby acting as a key benchmark for program directors when evaluating and comparing applicants. Since the transition of USMLE Step 1 to a pass/fail system in 2022, the scores achieved in the USMLE Step 2CK are now considered even more important in the application process^12,13^. Interestingly, Kracaw et al. showed that across different academic performance measures, the USMLE Step 1 score showed the highest correlation with USMLE Step 2CK scores. This significant correlation can be attributed to the similar question format between both examinations and the fundamental knowledge established through USMLE Step 1 which is essential to understand and anticipate clinical scenarios tested in USMLE Step 2CK^14^. Overall, USMLE Step 1 serves as the cornerstone for competitive scoring in USMLE Step 2CK.

However, despite its significance, there remains a knowledge gap to this day investigating the ChatGPT’s performance on USMLE Step 1 test questions in a large-scale study. Further, a more comprehensive viewpoint of ChatGPT’s performance on USMLE Step 1 test questions including subject- and subspeciality-specific performance is still warranted to gain a more in-depth understanding of ChatGPT’s strengths and limitations for medical education in general and medical test-taking specifically.

Herein, we aimed to assess ChatGPT’s performance on USMLE Step 1 practice test questions based on 2,377 Amboss USMLE Step 1 style practice questions. Ultimately, this line of research may serve as a reference work on how to integrate AI and ChatGPT into medical education while shielding USMLE Steps against AI cheating.

## Methods

### Question bank access and ChatGPT data entry

Between June 5, 2023, and June 12, 2023, we utilized the Amboss question bank (New York, NY, USA) and collected 2,729 USMLE Step 1 style practice questions. Prior to commencing the study, we obtained official permission from Amboss (Amboss GmbH, Berlin, Germany) to use their USMLE Step 1 practice question bank for research purposes. The authors specifically chose the Amboss question bank since it has demonstrated its effectiveness in accurately mirroring the format and content of actual USMLE examination questions, thereby allowing to compare the test taking performance in the Amboss question bank with the actual USMLE examination^15^. Two examiners (M.A. and L.K.) randomly cross-checked the inputs to ensure that none of the answers were indexed on Google prior to June 12, 2023, which represents the latest accessible date in the ChatGPT training dataset.

All sample test questions underwent independent screening by four examiners (M.A., S.K., C.C.H., and L.K.), and any questions containing clinical images and photographs were excluded. After removing 352 image-based questions, we classified the remaining 2,377 test questions based on their medical specialty, using the categorization provided by Amboss. The provided categorizations are summarized in Table 1. All questions included in this study were in the format of multiple-choice single-answer style questions.

**Table 1:** Test question categories included in this study ranked by total number and ChatGPT’s performance.

Ranking	Question category	Number of Questions [%]	Ranking	Question category	Correct responses [%]
1	General Principles, Foundational Science	297 [12.5%]	1	Behavioral Health	77.9%
2	Nervous System & Special Senses	263 [11.1%]	2	Skin & Subcutaneous Tissue	66.7%
3	Cardiovascular System	208 [8.8%]	3	Respiratory System	63.0%
4	Gastrointestinal System	191 [8.0%]	4	Social Sciences	61.1%
5	Multisystem Processes and Disorders	175 [7.4%]	5	Immune System	60.9%
6	Blood & Lymphoreticular	154 [6.5%]	6	Nervous System & Special Senses	60.8%
7	Respiratory System	146 [6.1%]	7	Male Reproductive System	57.9%
8	Endocrine System	137 [5.8%]	8	General Principles, Foundational Science	57.6%
9	Renal & Urinary Systems	128 [5.4%]	9	Musculoskeletal System	57.4%
10	Immune System	110 [4.6%]	10	Pregnancy & Childbirth	57.1%
11	Musculoskeletal System	108 [4.5%]	11	Female Reproductive System	56.6%
12	Behavioral Health	95 [4.0%]	12	Gastrointestinal System	54.5%
13	Female Reproductive System	76 [3.2%]	13	Biostats & Epidemiology	50.7%
14	Skin & Subcutaneous Tissue	72 [3.0%]	14	Renal & Urinary Systems	48.4%
15	Biostats & Epidemiology	69 [2.9%]	15	Multisystem Processes and Disorders	46.9%
16	Pregnancy & Childbirth	56 [2.4%]	16	Blood & Lymphoreticular	45.5%
17	Social Sciences	54 [2.3%]	17	Endocrine System	45.3%
18	Male Reproductive System	38 [1.6%]	18	Cardiovascular System	44.7%
Total		2377 [100.0%]	Total		55.2%

To assess the difficulty of each test question, we designed a difficulty classification based on the average performance of the human user peer group:: Category 1 (“Very Easy”) with an average performance of 81–100% of , Category 2 (“Easy”) with an average performance of 61–80%, Category 3 (“Intermediate”) with an average performance of 41–60%, Category 4 (“Difficult”) with an average performance of 21–40%, and Group 5(“Very Difficult”) with an average performance of 0–20%.This linear classification system is based on the performance of all user responses to that respective question on Amboss^16^.

To investigate whether the accuracy of ChatGPT's responses varied across different patient age groups, we analyzed its performance across the following age ranges: 1–12 month(s), 1–10 year(s), 11–20 years, 21–30 years, 31–40 years, 41–50 years, 51–60 years, 61–70 years, 71–80 years, 81–90 years, and 91–100 years. Furthermore, we analyzed the question stems for specific signaling words, such as "most likely/probable cause," "most appropriate treatment," "most likely/probable diagnosis," as well as diagnostic methods and provided patient information including "ECG," "Laboratory values," "MRI," "CT," "Ultrasound," "Culture," "Serology," "Biopsy," "Histology," "Endoscopy," "Emergency Department," "Alcohol Abuse," "Nicotine Abuse," and "Illicit Drugs." This analysis aimed to identify any differences in accuracy based on the presence of these specific factors.

One examiner (M.A.) manually inputted the test questions into ChatGPT 3.5 (OpenAI, San Francisco, CA, USA). The USMLE Step 1 practice questions from the Amboss question bank were transcribed precisely, maintaining the original question text and answer choices. To ensure the integrity of ChatGPT's performance, the authors intentionally refrained from introducing any additional prompts, thereby minimizing the potential for systematic errors. For each question, a new chat session was initiated in ChatGPT to minimize the impact of memory retention bias. An example of a standard test question is as follows:

*„A 27-year-old woman visits the clinic seeking counseling before planning a pregnancy. She mentions that her friend recently gave birth to a baby with a neural tube defect, and she wants to reduce the risk of having a child with the same condition. The patient has no significant medical history and is not taking any medications. Physical examination reveals no abnormalities. What is the most appropriate recommendation for this patient regarding vitamin supplementation that acts as a cofactor in which of the following processes?“.*

- (A) Synthesis of nucleotides
- (B) Gamma glutamate carboxylation of proteins
- (C) Neutralizing free radicals
- (D) Transketolation
- (E) Breakdown of triglycerides through lipolysis

The answers provided by ChatGPT were documented and inputted into the online platform for the respective Amboss USMLE Step 1 practice question. Subsequently, information regarding the accuracy of these responses, along with the percentage of the user peer group having selected the correct answer (or incorrect answer if ChatGPT chose incorrectly), were systematically gathered and recorded in a separate data spreadsheet. The user peer group is defined as all registered users on the Amboss question bank responding to the respective question.

### Statistical analysis

Differences between question style and categories were determined using Pearson’s chi-square test. Bivariate correlation analysis between ChatGPT performance and test question length and difficulty relied on the calculation of Spearman’s correlation coefficient (r_s_). Statistical analysis was conducted with IBM SPSS Statistics 27, and a two-tailed p-value of ≤ 0.05 was deemed to indicate statistical significance.

## Results

### General test question characteristics and performance statistics

ChatGPT answered 98.9% (2351/2377) of USMLE Step 1 practice questions. The overall accuracy of ChatGPT was 55.8% (1312/2351 questions). The mean length of the included practice questions was 762.1 ± 211.3 characters [range: 283 – 2,168] and 105.5 ± 36.2 words [range: 33–445]. Test question length and ChatGPT’s performance were not significantly correlated for neither character count (r_s_ = − 0.010; p = 0.627) nor word count (r_s_ = 0.024; p = 0.239). An inverse correlation was found for question length and difficulty (as defined by the difficulty classification based on the performance of the human user peer group) with r_s_ = − 0.089 (character count) and r_s_ = -0.131 (word count) with both p < 0.001. Further information on the characteristics of the included practice questions is summarized in Supplemetary Tables 1–4.

### ChatGPT’s performance by question category/medical specialty

ChatGPT achieved the best performance in the “Behavioral Health” category with 77.9% (74/95 questions) and the worst performance in the “Cardiovascular System” category with 44.7% (93/208 questions). Further information on the number of questions per category and ChatGPT’s respective performance is summarized in Table 1.

### ChatGPT’s performance by test question difficulty

**Figure 1:**
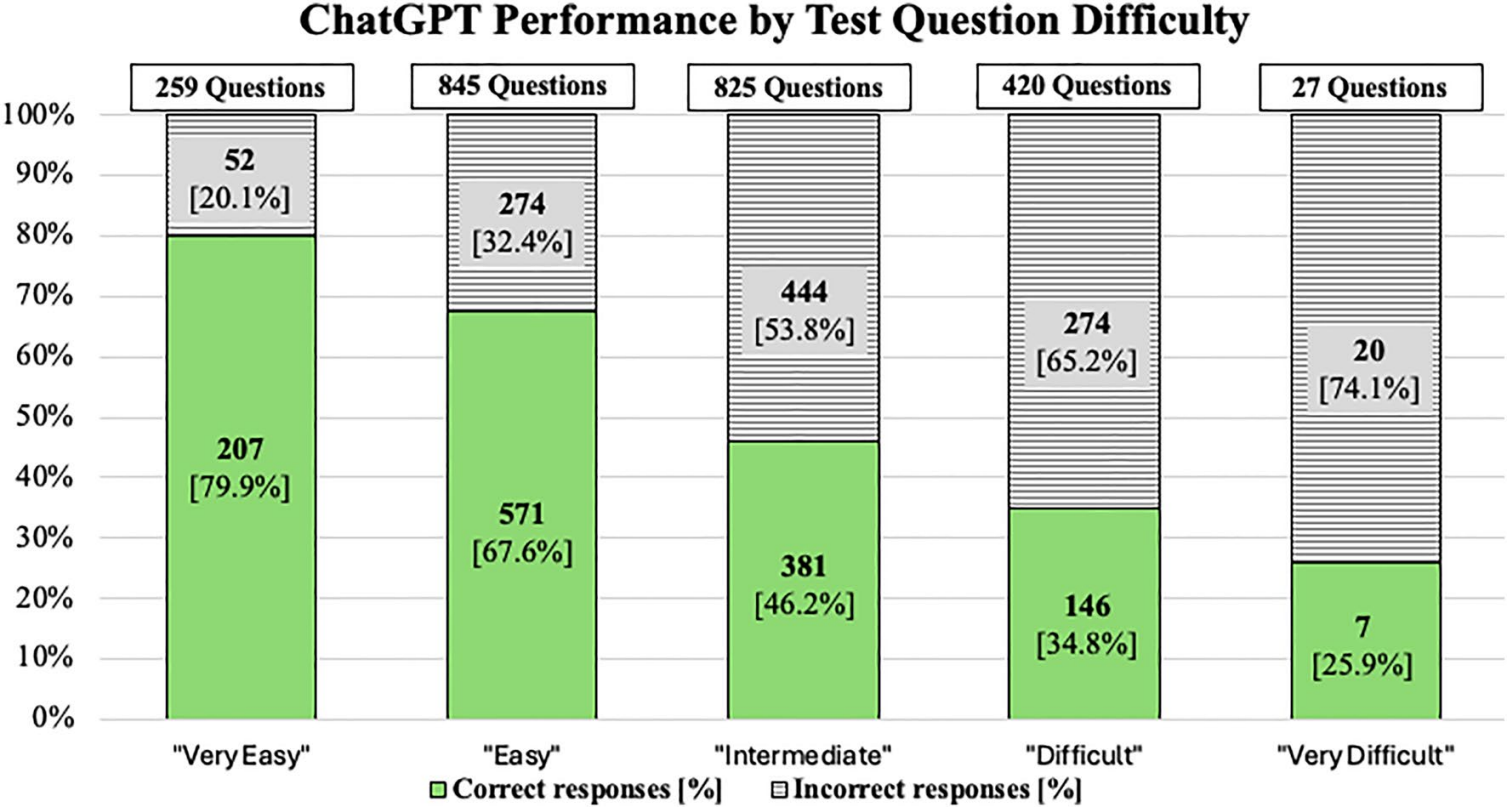
Performance of ChatGPT as percentage of correctly answered test questions, stratified by level of test question difficulty. (i.e., difficulty classification based on the performance of the human user peer group).

Test question difficulty (as defined by the difficulty classification based on the performance of the human user peer group) and ChatGPT’s performance showed a significant inverse correlation with r_s_ = -0.306 and p < 0.001. The average performance of ChatGPT versus the human Amboss user peer group were: 79.9% versus 87.0% (Category 1), 67.6% versus 70.1% (Category 2), 46.2% versus 50.9% (Category 3), 34.8% versus 33.4% (Category 4), and 25.9% versus 16.3% (Category 5), respectively. ChatGPT’s performance stratified by test question difficulty is visualized in Fig. 1.

### ChatGPT’s performance by signaling words/phrases

**Figure 2:**
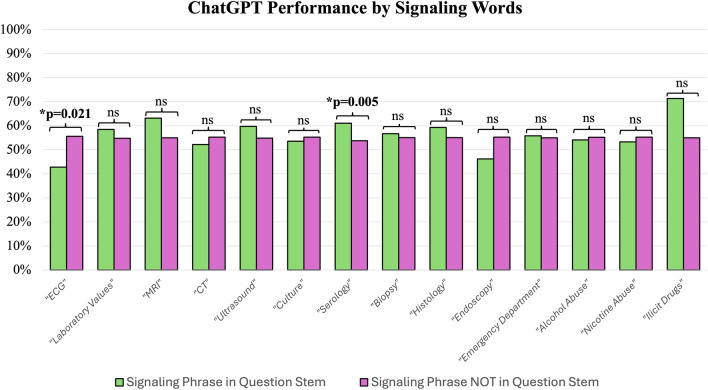
Paired bar graphs showing the performance of ChatGPT, stratified by the presence or absence of specific signaling words in the question stem.

**Figure 3:**
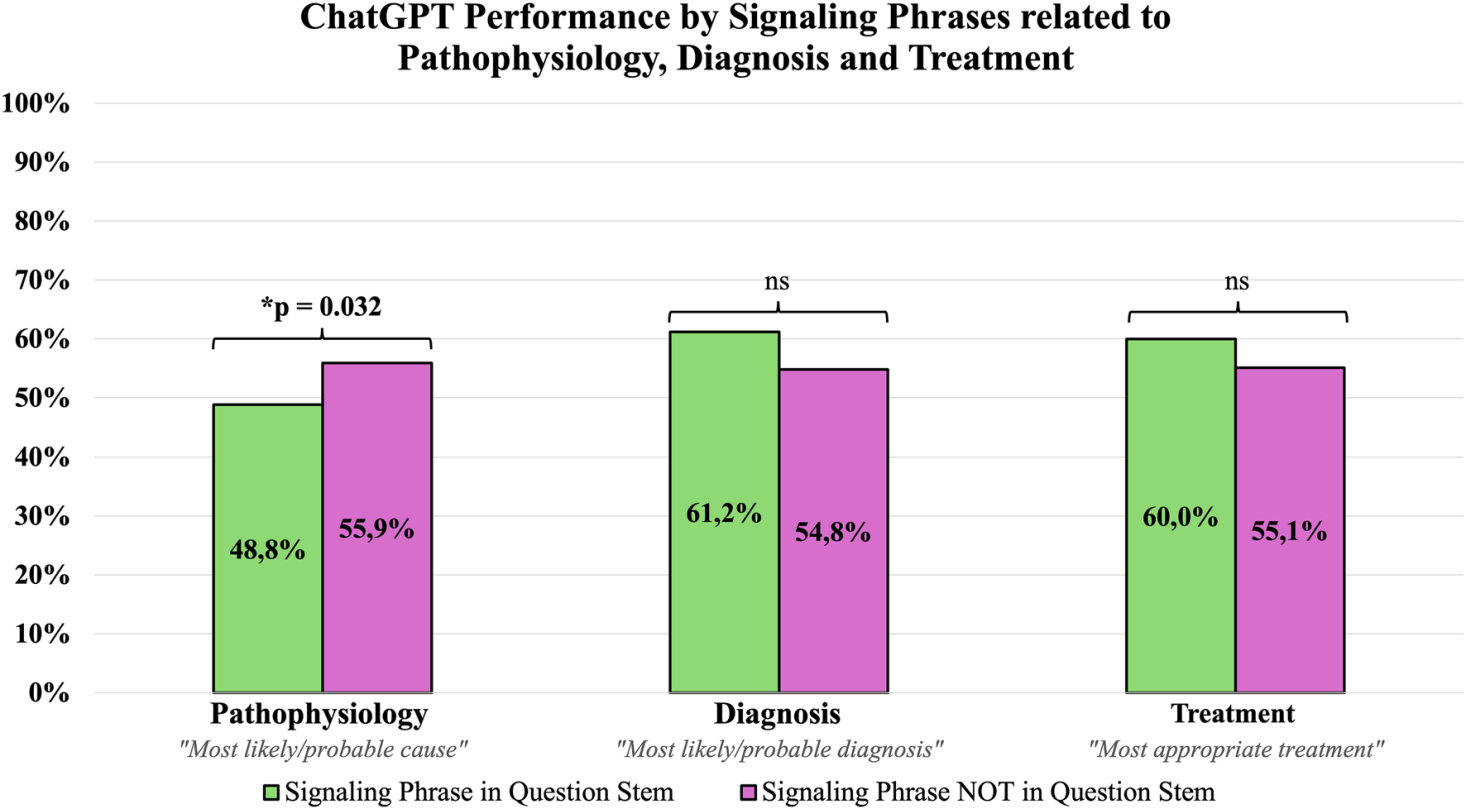
Paired bar graphs showing the performance of ChatGPT, stratified by the presence or absence of specific signaling phrases.

A statistically significant difference was found for test questions with and without the pathophysiology-related signaling phrase “Most likely/probable cause” (44.1% vs 51.2%), with p = 0.032. ChatGPT performed significantly worse when answering ECG-related test questions compared to non-ECG-related test questions (42.9% vs. 55.6%), with p = 0.021. Of note, ChatGPT’s performance was significantly better for serology-related questions (61.1% vs. 53.8%), with p = 0.005. ChatGPT’s test performance by signaling words and phrases are shown in Figs. 2 and 3, respectively.

### ChatGPT’s performance by patient age (in question stem)

**Figure 4:**
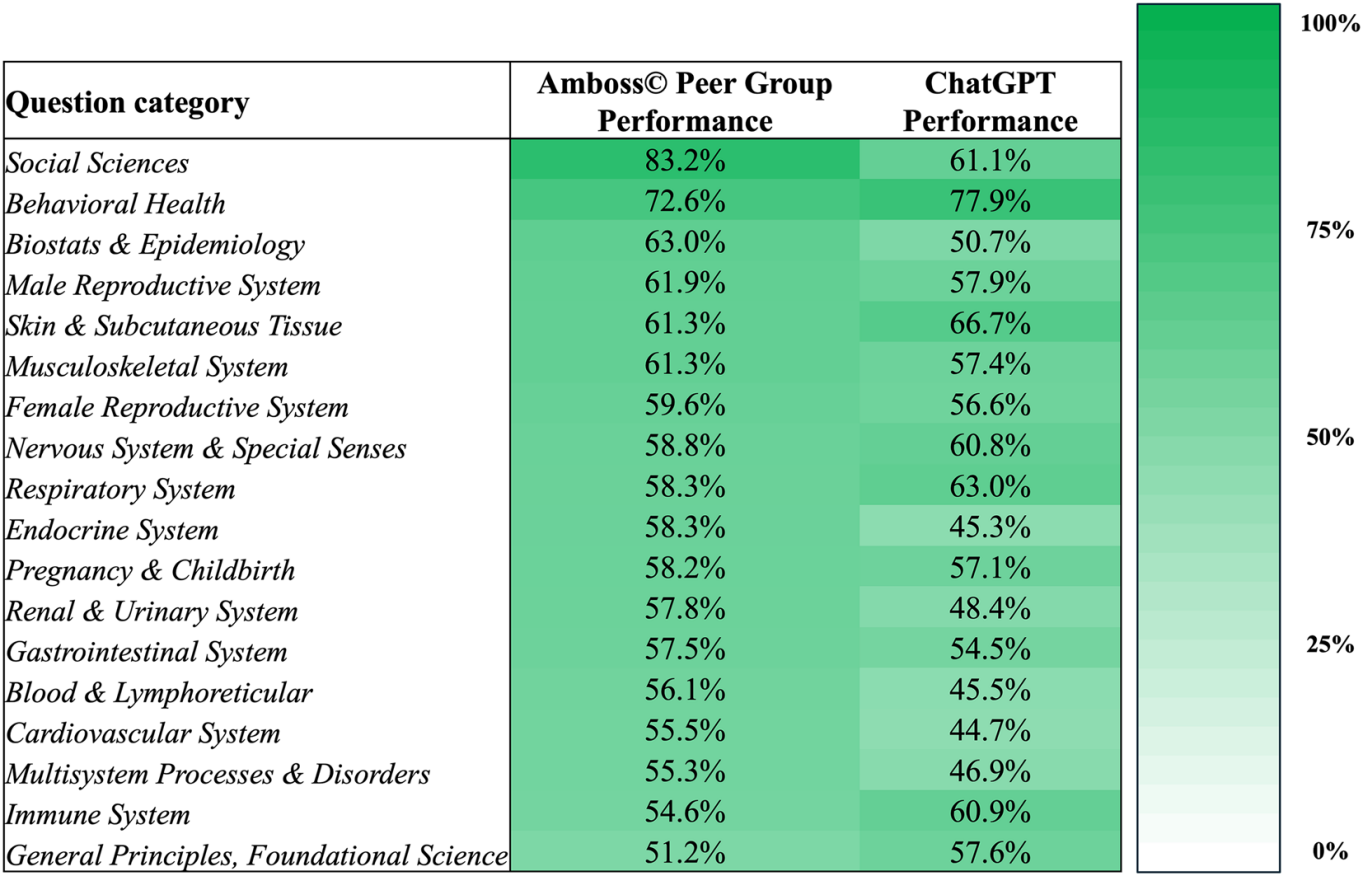
Heatmap comparing the performance of the Amboss user peer group and ChatGPT, stratified by patient age indicated in the question stem.

ChatGPT achieved the best performances for questions in which the patient belonged to the youngest age groups: “1–12 months” with 56.3% and “1–10 years” with 56.7% while the worst performances were achieved for questions in which the patient belonged to the oldest age groups analyzed in our study: “61–70 years” with 53.6% and “71–80 years” with 45.8%. Further information on ChatGPT’s performance for specific patient age categories is summarized in Fig. 4.

### ChatGPT’s performance compared to the human Amboss user peer group

**Figure 5:**
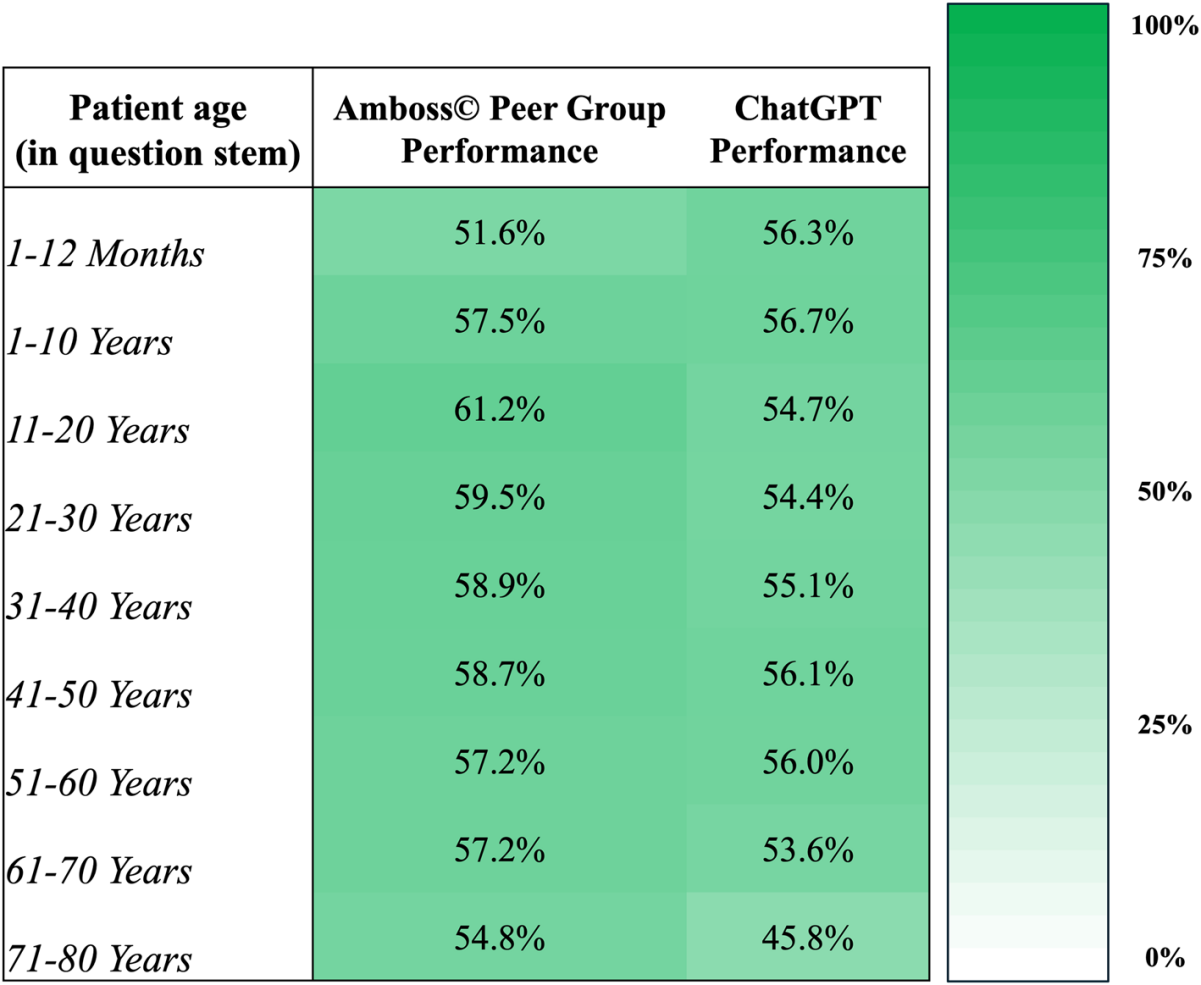
Heatmap comparing the performance of the Amboss user peer group and ChatGPT, stratified by question categories.

The overall performance of the human Amboss user peer group was 58.2%. Performance comparisons between the Amboss user peer group and ChatGPT sorted by patient age as indicated in the question stem and question category are illustrated in Figs. 4 and 5, respectively.

## Discussion

In the present study, the authors endeavoured to examine the proficiency of AI-powered chatbots, specifically ChatGPT, in accurately responding to practice questions for the USMLE Step 1 examination. The evaluation of such models holds significant importance as an accurate response to these examination questions necessitates the application of precise knowledge within a clinical context, thereby incorporating elements of integrative reasoning.

Our findings demonstrate that ChatGPT achieved an accuracy of 55.8%, approaching the passing threshold of approximately 60%. This is congruent with extant literature wherein ChatGPT’s performance hovers around this benchmark. Notably, Kung et al. also reported an identical accuracy of 55.8% for USMLE Step 1 questions^10^. Our study, however, bolstered this finding by employing a nearly 20-fold larger sample size, comprising 2,377 questions as opposed to the 119 questions used by Kung et al. In a study by Gilson et al., the accuracy rates reported were 44.0% and 64.4% for Amboss Step 1 and NBME Free Step 1, respectively, based on smaller sample sizes of 100 and 87 questions, respectively^17^. The discrepancies in accuracy rates in the latter study can be attributed to the limited sample sizes, which constrain the reliability of extrapolating ChatGPT's performance. These results need to be viewed in the context of the USMLE examination format containing only single-answer format questions. ChatGPT’s performance around the 60% mark – as demonstrated in our study—is in line with various previous studies also having included multiple-choice single-answer format questions for their analysis^10,17–21^. Conversely, Alfertshofer et al. were able to show in their multinational comparison of ChatGPT’s performance on medical state examinations that the chatbot scored significantly worse in multiple-choice question formats (i.e., French medical state examination) with an accuracy of 22%. This significant discrepancy underlines a key weakness of ChatGPT: Navigating multiple-choice questions and allowing for more than one correct answer per question. The underperformance in multiple-choice question formats might stem from the necessity of a deeper, more nuanced understanding of how different answer choices compare to each other and where the cut-off whether an answer choice is correct or incorrect should be drawn. This contrasts with the multiple-choice single-answer question format, where the process of answer selection is thought to be more straightforward due to the selection of only one instead of multiple answer choices. Hence, in other words, ChatGPT might exhibit greater proficiency to “find the most correct answer” rather than to “find all correct answers”.

Intriguingly, our study also illuminated a significant correlation between the performance of the human Amboss user peer group and ChatGPT’s performance: Test question difficulty (as defined by the difficulty classification based on the performance of the human user peer group) and ChatGPT’s performance is negatively correlated with r_s_ = -0.306; p < 0.001. Knoedler et al. reported an overall performance of ChatGPT with 56.9% on USMLE Step 3 preparation questions in a sample size of 1,840 questions^18^. Albeit being small, the difference in overall performance between USMLE Step 1 and Step 3 showed that ChatGPT found the former more challenging. This mirrors performance patterns on the actual USMLE examination: According to the official annual USMLE performance statistics for the year 2023, the USMLE Step 1 examination is consistently regarded as more arduous, with a pass rate of approximately 80% across all test-takers, encompassing candidates from both US/Canadian and Non-US/Canadian schools, in contrast to the notably higher pass rate of approximately 94% observed for USMLE Step 3. This parallelism reflects the underlying architecture of ChatGPT, which is a deep neural network trained on extensive text datasets, primarily derived from human-generated content. Consequently, ChatGPT inherits human knowledge, including areas of difficulty and propensities for errors. Therefore, the interplay between humans and ChatGPT transcends the simplistic dichotomy of “Man vs Machine,” and is more aptly characterized as a “Machine Predicated on Human Cognition.” The inability to surpass average human knowledge might be regarded as a weakness of large language models such as ChatGPT based on previously generated content by humans. However, it can also be leveraged – especially in the educational context—as ChatGPT and other AI-powered technologies can be employed as “diagnostic tools” to identify areas of difficulty within medical education and training. An in-depth large-scale analysis of error patterns and challenges encountered by AI in simulations of examinations like the USMLE can help educators, test writers and curriculum developers gain insights into which topics may require more focused instructional strategies, potentially resulting in more targeted educational approaches and higher overall passing rates in challenging examinations such as USMLE Step 1.

Further analysis of the interplay between human and ChatGPT accuracies, stratified by question difficulty, reveals a precipitous decline in the accuracy of correct responses with increasing difficulty for both cohorts. For questions of lower difficulty (i.e. difficulty category 1 “very easy”, category 2 “easy”, and category 3 “intermediate), ChatGPT’s performance was inferior to that of the human user peer group. Interestingly, this trend reverses with questions of higher difficulty (i.e., difficulty category 4 “difficult”, and category 5 “very difficult), where ChatGPT demonstrates a comparable and superior performance, respectively. This observation is somewhat counterintuitive given the above postulated hypothesis that ChatGPT’s performance would parallel human tendencies. One potential explanation for this observation lies in the questions’ design. Simpler questions might align more closely with common human knowledge or intuition, while more difficult questions might more often incorporate scenarios specifically designed to confuse test-takers, leveraging human cognitive biases or emotional responses. Due to its logic-based algorithmic approach of ChatGPT, it remains unaffected by psychological factors often influencing human decision-making as it analyzes and processes information in a structured manner. Future research will need to further expand on this assumption to see if it generally holds true.

Interestingly, ChatGPT achieved its best performances in the youngest age groups (i.e., “1–12 months” and “1–10 years”) while its worst performances were found in the oldest age groups (i.e., “61–70 years” and “71–80 years”) analyzed in our study. Possible explanations for this performance difference might be found in a) the medical conditions typically encountered in these age groups and b) the volume and quality of training data ChatGPT was exposed to during its development. It might be speculated that the enhanced performances in the younger age groups are due to a larger volume and more standardized nature of internet-sourced health-related information mainly focussing on well-documented developmental stages and common pediatric conditions. Conversely, the poorer performance in older age groups might be drawn back to the complex interplay and intricate nature of various health conditions often influencing each other with increasing age as well as ChatGPT’s smaller exposure to geriatric health problems discussed online. Such disparity in training data could ultimately result in ChatGPT’s variable performance across different age categories, necessitating further investigation into the volume and quality of ChatGPT's training sources.

When taking a closer look at certain signal words contained in the question stem, we found that ChatGPT performed significantly worse when answering ECG-related questions with a performance of 42.9% in ECG-related questions versus 55.6% non-ECG-related test questions, with p = 0.021. On the other hand, ChatGPT was able to outperform human test-takers for questions on serological tests with a performance of 61.1% in serology-related questions versus 53.8% in non-serology-related questions, with p = 0.005. These novel findings align with ChatGPT’s strength profile. Different groups have underscored ChatGPT’s capabilities when analyzing numeric datasets^22–24^. Hence, a potential explanation for ChatGPT’s improved performance is the nature of serological test questions commonly including multiple numeric values, thereby allowing for an objective analysis by ChatGPT. In contrast, ECG-related test questions oftentimes contain additional written-out information (e.g., ST-segment elevation, flattened T-wave) relevant to choose the correct answer. These elements introduce a layer of complexity and abstraction that exceeds mere objective numerical analysis. Consequently, the performance difference observed in our study can be attributed to the nature of ECG-related questions which require a nuanced understanding and integration of abstract descriptive findings to arrive at the correct answer. Overall, these findings reinforce the importance of teaching abstraction capability and comprehensive understanding in medical schools.

### Limitations

This study however is not without limitations. Firstly, due to its widespread availability and significantly larger userbase, the authors decided to employ version 3.5 to test ChatGPT’s performance in a large sample of 2,377 USMLE Step 1 style practice questions. However, the findings presented herein have to be reevaluated for future ChatGPT versions. Secondly, future studies should focus on an in-depth comparison between different large-language models and chatbots to gain further insights into their respective strengths and weaknesses when providing healthcare information to different stakeholders (e.g., medical student, doctor, patient) in different contexts. Also, additional question banks, as well as image-based test questions can be included for future studies in this field. Thirdly, the lack of a another subclassification of question types—for instance in factoid, procedural, causal, and comparative questions—limits the study's ability to fully explore ChatGPT's nuanced performance across diverse medical examination questions. Such specificity could reveal critical insights into ChatGPT’s strengths and weaknesses in processing and responding to complex medical questions in different test-taking contexts. Future studies focusing on this granularity might significantly enhance our understanding of AI’s role in medical education, particularly by identifying targeted areas for improvement in educational tools. Lastly, it is important to acknowledge the limitation stemming from our study's focus on text-based questions and the exclusion of artificially created queries designed to assess ChatGPT's ability to navigate different levels of question complexity and questioning techniques. This limitation may impact our findings’ generalizability to scenarios involving advanced test question formulation aimed at preventing AI-assisted cheating. Future research could benefit from incorporating these dimensions to fully ascertain ChatGPT's utility and limitations in medical education and examination contexts.

## Conclusion

ChatGPT’s test-taking performance based on 2,377 USMLE Step 1 questions was analyzed and it was found that ChatGPT carries the potential to outperform human examinees. Besides distinct weakpoints such as ECG-related test questions, ChatGPT demonstrated well-balanced test-taking performances similar to the average of human test-takers. Overall, this study reinforces the need for developing AI-proof exams and preventing AI-cheating.

### Supplementary Information

Supplementary Table 1.

Supplementary Table 2.

Supplementary Table 3.

Supplementary Table 4.

## Data Availability

The datasets used and/or analysed during the current study available from the corresponding author on reasonable request.
